# Association of lung health and cardiovascular health (Life’s Essential 8)

**DOI:** 10.3389/fmed.2025.1481213

**Published:** 2025-02-04

**Authors:** Wanjia Zhang, Menglong Zou, Junyao Liang, Dexu Zhang, Man Zhou, Hui Feng, Chusen Tang, Jie Xiao, Zhan Yi, Yin Xu

**Affiliations:** ^1^The First Hospital of Hunan University of Chinese Medicine, Changsha, Hunan, China; ^2^Hunan University of Chinese Medicine, Changsha, Hunan, China

**Keywords:** cardiovascular health, lung health, Life’s Essential 8, NHANES, cross-sectional study

## Abstract

**Background:**

Cardiorespiratory health issues often intersect. This study explored the relationship between lung health and the recently updated Cardiovascular Health Measure (Life’s Essential 8).

**Methods:**

Data from the 2007–2018 National Health and Nutrition Examination Survey (NHANES) were analyzed. Three cohorts were included: lung disease in the Main cohort (*N* = 9,772), lung function in the Spirometry sub-cohort (*N* = 3,896), and respiratory symptoms in the Respiratory Symptoms sub-cohort (Age > 40) (*N* = 3,449). LE8 scores were used as both continuous and categorical variables (0–49, 50–79, 80–100). Weighted multivariate regression analyses examined the correlations between LE8 and lung health, and weighted restricted cubic spline (RCS) regression analyzed potential non-linear relationships. Subgroup analysis was conducted to verify stability.

**Results:**

Overall lung health was better in the high LE8 group than in the low LE8 group. In the fully adjusted model, the high LE8 group had significantly lower odds of asthma (OR = 0.42; 95% CI = 0.29, 0.59) and chronic bronchitis (OR = 0.27; 95% CI = 0.15, 0.49) compared to the low LE8 group. Regarding lung function, each 10-point increase in LE8 was associated with an increase of 50 ml in FEV1 (Beta = 50; 95% CI = 32, 67) and 56 ml in FVC (Beta = 56; 95% CI = 32, 79). Among middle-aged and older adults (age > 40), the high LE8 group had significantly lower odds of respiratory symptoms, including cough (OR = 0.23; 95% CI = 0.12, 0.46), phlegm (OR = 0.42; 95% CI = 0.19, 0.90), and wheezing (OR = 0.29; 95% CI = 0.15, 0.54). RCS analyses demonstrated a non-linear negative correlation between LE8 and cough, sputum, and wheeze. Subgroup and sensitivity analysis suggested stability.

**Conclusion:**

Life’s Essential 8 (LE8) scores are positively associated with lung health in the US population. These findings provide a valuable reference for maintaining overall cardiorespiratory health.

## 1 Introduction

Lung health is a critical component of overall human health, encompassing various factors from respiratory symptoms and lung function to specific pulmonary diseases. Clinical manifestations of respiratory diseases typically include chronic cough, sputum production, and wheezing or whistling. These discomforts have a substantial impact on the quality of life. Pulmonary function tests, including forced vital capacity (FVC) and forced expiratory volume in one second (FEV1), are commonly used indicators of disease severity and progression (1). Additionally, chronic lung diseases affect approximately 454 million people worldwide, leading to nearly 4 million deaths annually (2). These pulmonary conditions impose a substantial burden on affected individuals and healthcare systems globally.

The heart and lungs are the primary organs of two vital systems in the body. Their risk factors partially overlap, leading to the frequent intersection of heart and lung health issues. Cardiovascular events often occur in patients with obstructive lung diseases, such as chronic obstructive pulmonary disease (COPD), particularly in the later stages (3). Both cardiovascular disease and asthma are associated with similar systemic chronic inflammatory factors (4). An observational research demonstrated a negative correlation between lung function and the incidence of stroke (5). A healthy lifestyle, encompassing a balanced diet, adequate sleep, and appropriate physical activity, has a substantial impact on lung health (6–10). Furthermore, metabolic factors, including lipid levels, blood glucose, and other metabolic parameters, are critical considerations in lung health, as metabolic abnormalities frequently influence the progression of chronic lung diseases (11–13). Therefore, a comprehensive indicator is essential for effective lung health management.

In 2022, the American Heart Association introduced a new cardiovascular health metric known as “Life’s Essential 8” (LE8) (14). This metric builds on its predecessor, Life’s Essential 7 (LE7), which has been recognized as a crucial primary prevention indicator within the healthcare system (15). LE8 enhances LE7 by incorporating sleep quality and redefining the algorithm used for assessment. Studies have demonstrated that LE8 not only improves the cardiovascular health of the general population but also benefits a range of chronic conditions, including cancer (16), chronic kidney disease (17), bowel health (18), depression (19), and endocrine disorders (20). LE8 consists of components related to a healthy lifestyle and health factors. The healthy lifestyle components include diet, physical activity, nicotine exposure, and sleep health. The health factors encompass body mass index (BMI), blood pressure, blood glucose levels, and cholesterol levels. By addressing these components, LE8 provides a comprehensive approach to promoting cardiovascular health and preventing chronic diseases.

The association of Life’s Essential 8 (LE8) with lung health has not been previously reported. To address this gap, we aimed to explore this association in a US population. We retrospectively analyzed data from NHANES participants over the past decade. Lung health was assessed at three levels: (1) respiratory symptoms; (2) pulmonary function tests; and (3) self-reported chronic lung disease. The findings of the research may improve our understanding of cardiopulmonary health as a whole, thereby facilitating multiple prevention strategies.

## 2 Materials and methods

### 2.1 Research design

The National Health and Nutrition Examination Survey (NHANES) is a major program for monitoring the health and nutritional status of the US population (21). It utilizes a complex, multistage sampling design that includes a wide range of participants from diverse population groups. NHANES collects data on demographics, dietary habits, medical history, and physical and laboratory assessments, providing an essential resource for tracking public health trends and developing health policy. All participants provide informed consent, and the survey protocol undergoes rigorous ethical review. Detailed methodology and data from NHANES are publicly available on the official website.

This study accounted for the variability of data from different cycles of the NHANES study. Data on lung function and respiratory symptoms were collected only during the three cycles from 2007 to 2012, with respiratory symptoms recorded only for participants over 40 years old. We included three groups in this study: the Main cohort, the Spirometry sub-cohort, and the Respiratory Symptoms sub-cohort.

**FIGURE 1:**
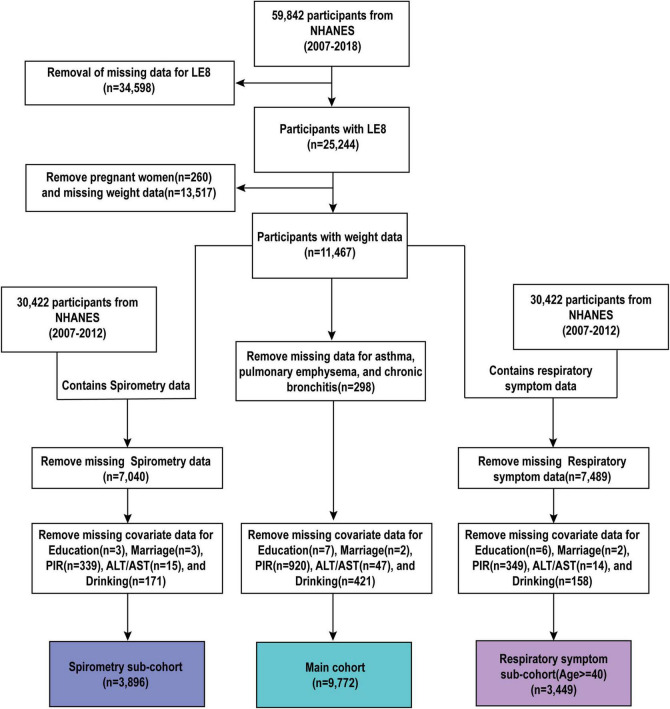
Flowchart of the study population.

Initially, we performed a primary screening to identify participants with available LE8 data, eligible data, and no pregnancies, totaling 11,467 participants. The Main cohort excluded participants with a history of asthma, chronic bronchial disease, and emphysema, resulting in a final sample size of 11,169 participants. The Spirometry sub-cohort excluded participants with missing FEV1, FVC, and FENO data, with a final total of 4,818 participants included. The Respiratory Symptoms sub-cohort excluded participants with missing respiratory symptoms data, resulting in a final total of 3,978 participants. Figure 1 illustrates the flowchart of the survey.

### 2.2 Assessment of Life’s Essential 8

According to the American Heart Association’s most recent cardiovascular health standards (14), Life’s Essential 8 (LE8) consists of four health factors (body mass index, lipids, blood glucose, and blood pressure) and four health behaviors (diet, physical activity, nicotine exposure, and sleep health). Based on previous research (14, 20), physical activity is typically assessed by the amount of time spent engaging in moderate or vigorous exercise per week, sleep health is evaluated by the average sleep duration per night, and nicotine exposure is evaluated based on smoking status and the duration since quitting.

Dietary indicators were assessed with reference to the Healthy Eating Index-2015 (HEI-2015) (22), which was calculated from information on dietary intake collected by questionnaires on two occasions over a 24-h period (23, 24). Supplementary Table 1 provides a detailed description of the HEI-2015. It assessed the diet of the US population in 13 different areas. The scores for the four health factors were assessed by the respective value ranges. Specific calculations of the LE8 can be viewed in the Supplementary Table 2. Data on smoking, sleep, physical activity, history of diabetes mellitus, and history of medication use were collected via questionnaire in the NHANES study. Physical examinations measured BMI and blood pressure, while laboratory tests assessed lipids, blood glucose, and glycosylated hemoglobin levels.

The scores for the eight indicators range from 0 to 100, with the total LE8 score being the average of the eight indicators. Following the recommendations of the American Heart Association and previous research, we categorized LE8 scores into three groups: 0–49 as low, 50–79 as moderate, and 80–100 as high (14, 20, 25). This classification was based on its practical application in clinical and public health contexts, facilitating the interpretation of results and identification of populations with differing cardiovascular health levels.

### 2.3 Assessment of lung health

Lung function was measured using the Ohio 822/827 Dry Rolled Sealed Volumeter. Lung capacity was typically measured in a standing position. During each maneuver, participants breathed deeply to fulfill their lungs with air before being directed to conduct a series of maximally powerful expiratory maneuvers (26). The relationship between LE8 and lung function was analyzed using FVC, FEV1, and the FEV1 to FVC ratio (27). Additionally, we included the fraction of exhaled nitric oxide (FENO) as a non-invasive marker of airway inflammation. The presence or absence of airway inflammation was categorized according to whether the FENO score was greater than 50 (28).

The assessment of Lung Diseases were based on questionnaire data. Participants were categorized as having asthma or not according to their responses to the question, “Have you been told by a doctor or other health professional that you have asthma?” Similarly, participants were categorized as having chronic bronchitis based on their “yes” or “no” responses to the question, “Have you been told by a doctor or other health professional that you have chronic bronchitis?” (29–32).

The respiratory symptoms questionnaire was administered to participants who were 40 years and older. The presence or absence of coughing symptoms was determined by participants’ responses of “yes” or “no” to the question, “Do you cough most days of the year for 3 consecutive months or more?” The presence or absence of sputum production was assessed with the question, “Do you bring up phlegm most days of the year for 3 consecutive months or more?” Wheezing or whistling in the chest was evaluated based on the participants’ responses to the question, “In the past 12 months, has there been wheezing or whistling in the chest?” Additionally, the presence or absence of hay fever was determined using the question, “Have you had a hay fever attack in the past 12 months?” (33, 34).

### 2.4 Covariate

Our analyses considered potential confounders, including age, race, sex, marital status, poverty income ratio (PIR), education, history of drinking, family history of asthma, and levels of alanine aminotransferase and aspartate aminotransferase. Education level was classified as < high school, high school, and > high school. Marital situation was classified as married, partnered, or alone. Race was categorized into five groups: Mexican American, non-Hispanic Black, non-Hispanic White, other Hispanic, and other race. PIR, an index used to measure household socioeconomic status, was categorized as < 1.5, 1.5–3.5, or > 3.5 (35). Alcohol consumption history was categorized based on the frequency of consumption as never, sometimes, and often. Family history of asthma was determined by responses to the question, “Do you have a close relative with asthma?”

### 2.5 Statistics analyses

Given the complex survey design of NHANES, including stratification, clustering, and oversampling of specific subpopulations, the analyses appropriately accounted for sample weights. The use of these weights ensures that the results are representative of the US population and reduces potential bias caused by unequal probabilities of selection and non-response. Continuous data were presented as weighted averages (standard deviations) and category variables as unweighted frequencies (weighted frequencies). The Kruskal–Wallis test was utilized for continuous variables and the chi-square test was employed for categorical variables to calculate between-group differences. Based on previous research experience and statistical analysis results (36, 37), we selected the following three models for regression analysis: Model 1: Non-adjusted; Model 2: Adjusted for key demographic and socioeconomic variables (age, sex, race, marital status, education, and PIR); Model 3: Further adjusted for additional variables (family history of asthma, drinking status, and ALT/AST). Dichotomous variables were assessed using multifactor logistic regression, while continuous variables were analyzed using linear models. The potential non-linear relationship between LE8 and lung health was examined using weighted restricted cubic spline (RCS) regression with 4 knots positioned at the 5th, 35th, 65th, and 95th percentiles of the exposure variable. RCS was selected for its ability to model non-linear relationships flexibly without requiring a specific parametric form. Compared to polynomial regression, RCS better captures localized changes, minimizes overfitting, and offers intuitive visualizations for easier interpretation.

The Subgroup analyses were stratified by age, sex, marital status, race, educational background, and income, with interaction tests used to assess the presence of dependent subgroups. As They are associated with access to healthcare, lifestyle factors, and overall health status. And interaction tests were used to assess whether the observed effects varied significantly across these subgroups, helping to identify potential differences in how the exposure impacts different populations. By identifying these differences, we can help inform more tailored and effective health policies that address the specific needs of diverse groups.

In the sensitivity analysis, we used the JOMO package designed for survey data to perform multiple imputation for the missing data (38, 39). To ensure stochastic independence, the Gibbs sampling algorithm was applied, generating 5 imputed datasets after a burn-in of 500 iterations and 100 updates. The method has been validated in previous studies (40). Regression analyses were subsequently repeated on the imputed datasets to assess the robustness of the results. R software version 4.2.3 was used for all analyses.

## 3 Result

### 3.1 Basic features of participants

A whole of 9,772 individuals were included in the Main cohort (Table 1), with 1,776 participants having low LE8 scores (0–49), 6,787 participants with intermediate LE8 scores (50–79), and 1,209 participants with high LE8 scores (80–100). Among the participants, 1,435 (weighted 15%) self-reported asthma, and 595 (weighted 5.7%) self-reported chronic bronchitis. The prevalence of asthma and chronic bronchitis was significantly lower in the high and intermediate LE8 groups compared to the low LE8 group. Participants with higher LE8 scores tended to be younger, non-Hispanic White, married, with higher educational attainment, and higher income levels.

**TABLE 1:** Main cohort participant characterization based on Life’s Essential 8 (LE8) scores.

Characteristic	Overall, *N* = 164,957,160	Low LE8, *N* = 1,776 (15%)	Mediate LE8, *N* = 6,787 (70%)	High LE8, *N* = 1,209 (15%)	*P*-value
Age, years, mean (SD)	48.13 (16.80)	53.56 (14.58)	48.63 (16.91)	40.50 (15.65)	< 0.001
Race (%)					< 0.001
Mexican American	1,403 (7.6%)	256 (8.2%)	968 (7.4%)	179 (8.0%)	–
Other Hispanic	965 (5.0%)	163 (4.8%)	679 (5.0%)	123 (5.4%)	–
Non-Hispanic white	4,521 (70%)	744 (65%)	3,187 (71%)	590 (74%)	–
Non-Hispanic black	1,863 (10%)	509 (17%)	1,242 (9.9%)	112 (4.8%)	–
Other race	1,020 (6.8%)	104 (4.7%)	711 (7.0%)	205 (8.0%)	–
Sex (%)					< 0.001
Male	4,736 (49%)	836 (45%)	3,412 (51%)	488 (41%)	
Female	5,036 (51%)	940 (55%)	3,375 (49%)	721 (59%)	
Education (%)					< 0.001
<High school	2,102 (14%)	560 (24%)	1,403 (14%)	139 (6.1%)	–
High school	2,218 (23%)	484 (31%)	1,557 (23%)	177 (14%)	–
> High school	5,452 (63%)	732 (45%)	3,827 (63%)	893 (80%)	–
Marital status (%)					0.004
Married	5,175 (57%)	836 (49%)	3,680 (58%)	659 (58%)	–
Partner	767 (8.0%)	153 (9.6%)	519 (7.8%)	95 (7.4%)	–
Alone	3,830 (35%)	787 (41%)	2,588 (34%)	455 (35%)	–
Family income (%)					< 0.001
Low	4,275 (31%)	1,015 (46%)	2,843 (30%)	417 (24%)	–
Medium	2,437 (26%)	432 (27%)	1,699 (26%)	306 (25%)	–
High	3,060 (43%)	329 (27%)	2,245 (44%)	486 (52%)	–
ALT (U/L)	24.99 (16.95)	27.30 (20.20)	25.26 (16.81)	21.50 (13.18)	< 0.001
AST (U/L)	24.97 (16.60)	25.76 (15.51)	25.06 (17.77)	23.77 (11.02)	0.074
Drinking (%)					< 0.001
Never	3,037 (25%)	675 (35%)	2,058 (25%)	304 (19%)	–
Sometimes	4,428 (50%)	654 (40%)	3,112 (50%)	662 (61%)	–
Often	2,307 (25%)	447 (26%)	1,617 (25%)	243 (20%)	–
Asthma					< 0.001
No	8,337 (85%)	1,420 (79%)	5,830 (86%)	1,087 (89%)	–
Yes	1,435 (15%)	356 (21%)	957 (14%)	122 (11%)	–
Chronic bronchitis					< 0.001
No	9,177 (94%)	1,588 (90%)	6,401 (94%)	1,188 (98%)	–
Yes	595 (5.7%)	188 (10%)	386 (5.7%)	21 (1.7%)	–
LE8	64.75 (13.98)	42.06 (6.36)	65.03 (8.06)	85.45 (4.69)	< 0.001
HEI-2015 diet score					< 0.001
Low (0–49)	4,834 (51%)	1,282 (76%)	3,298 (52%)	254 (21%)	–
Moderate (50–79)	2,445 (24%)	350 (17%)	1,783 (26%)	312 (26%)	–
High (80–100)	2,493 (25%)	144 (6.9%)	1,706 (22%)	643 (53%)	–
Physical activity score					< 0.001
Low (0–49)	5,813 (56%)	1,491 (83%)	3,998 (56%)	324 (27%)	–
Moderate (50–79)	220 (2.5%)	23 (1.3%)	165 (2.7%)	32 (2.9%)	–
High (80–100)	3,739 (42%)	262 (16%)	2,624 (41%)	853 (70%)	–
Sleep health score					< 0.001
Low (0–49)	1,692 (14%)	655 (34%)	984 (13%)	53 (3.1%)	–
Moderate (50–79)	2,012 (19%)	408 (23%)	1,425 (20%)	179 (14%)	–
High (80–100)	6,068 (66%)	713 (44%)	4,378 (67%)	977 (83%)	–
Nicotine exposure score					< 0.001
Low (0–49)	2,062 (21%)	799 (47%)	1,241 (19%)	22 (2.3%)	–
Moderate (50–79)	2,229 (23%)	432 (24%)	1,620 (25%)	177 (17%)	–
High (80–100)	5,481 (56%)	545 (29%)	3,926 (56%)	1,010 (81%)	–
BMI score					< 0.001
Low (0–49)	3,830 (38%)	1,270 (73%)	2,490 (38%)	70 (5.4%)	–
Moderate (50–79)	3,225 (33%)	361 (19%)	2,514 (37%)	350 (28%)	–
High (80–100)	2,717 (29%)	145 (7.5%)	1,783 (26%)	789 (66%)	–
Blood lipids score					< 0.001
Low (0–49)	3,105 (31%)	963 (57%)	2,054 (31%)	88 (7.3%)	–
Moderate (50–79)	2,256 (24%)	334 (19%)	1,718 (26%)	204 (17%)	–
High (80–100)	4,411 (45%)	479 (24%)	3,015 (42%)	917 (76%)	–
Blood glucose score					< 0.001
Low (0–49)	1,660 (12%)	748 (37%)	904 (9.6%)	8 (0.5%)	–
Moderate (50–79)	2,440 (21%)	606 (35%)	1,741 (21%)	93 (6.6%)	–
High (80–100)	5,672 (67%)	422 (28%)	4,142 (69%)	1,108 (93%)	–
Blood pressure score					< 0.001
Low (0–49)	2,472 (21%)	956 (50%)	1,484 (19%)	32 (2.1%)	–
Moderate (50–79)	2,896 (30%)	483 (29%)	2,215 (33%)	198 (17%)	–
High (80–100)	4,404 (49%)	337 (22%)	3,088 (48%)	979 (81%)	–

BMI, body mass index; ALT, alanine aminotransferase; AST, aspartate aminotransferase.

A whole of 3,896 individuals were included in the Spirometry sub-cohort (Supplementary Table 3), with a mean FEV1 of 3,251 ml, a mean FVC of 4,176 ml, and a mean FEV1/FVC ratio of 77.92%. The Respiratory Symptoms sub-cohort included 3,449 participants (Supplementary Table 4). Among these, 395 participants (weighted 11%) self-reported coughing, 351 participants (weighted 9.2%) reported bringing up phlegm, 490 participants (weighted 14%) reported wheezing or whistling in the chest, and 606 participants (weighted 22%) reported episodes of hay fever. Detailed information about the three sample groups is shown in Table 1, Supplementary Tables 3, 4.

**FIGURE 2:**
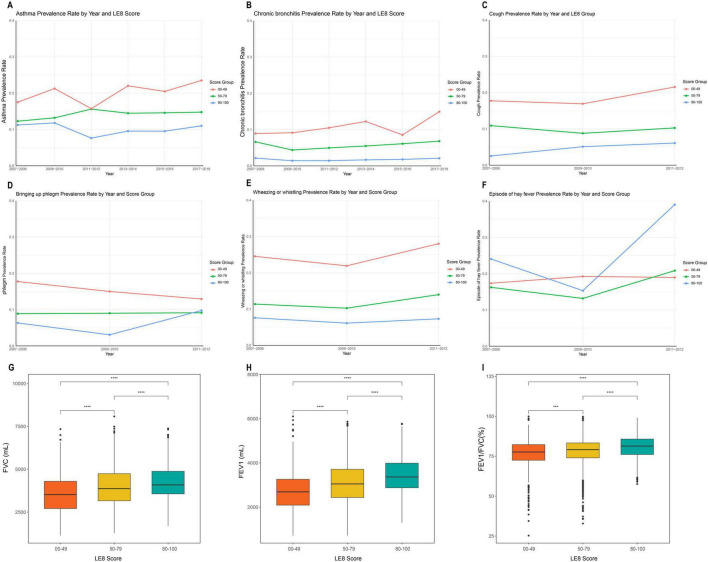
Flowchart of the study population. **(A)** Prevalence of asthma in each cycle after grouping by Life’s Essential 8 (LE8); **(B)** Prevalence of chronic bronchitis in each cycle after grouping by LE8; **(C)** Incidence of cough in each cycle after grouping by LE8; **(D)** Incidence of phlegm in each cycle after grouping by LE8; **(E)** Incidence of Wheezing or whistling in each cycle after grouping by LE8; **(F)** Incidence of hay fever in each cycle after grouping by LE8; **(G)** Comparison of forced vital capacity (FVC) between LE8 groups; **(H)** Comparison of forced expiratory volume in one second (FEV1) between LE8 groups; **(I)** Comparison of FEV1/FVC (%) between LE8 groups. ***P < 0.001; ****P < 0.0001.

Figure 2 illustrates the higher prevalence of asthma (A) and chronic bronchitis (B) in the low LE8 group across each cycle after stratification by LE8 scores. The prevalence of cough (C), sputum production (D), and wheezing (E) in each LE8 group is also depicted in Figure 2, indicating that the prevalence of pulmonary symptoms was significantly lower in the high LE8 group compared to the low LE8 group. Figure 2F shows the incidence of hay fever in each LE8 group. Additionally, Figure 2 presents the distribution of FEV1 (G), FVC (H), and the FEV1/FVC ratio (I) in each LE8 group, with *t*-tests conducted between groups. The results clearly demonstrate that lung function in the high LE8 group was superior to that in the low LE8 group.

### 3.2 Relationship between LE8 score and lung health

As shown in Table 2, weighted logistic regression revealed a significant negative association between LE8 scores and the prevalence of asthma and chronic bronchitis. In model 3, compared with the low LE8 group, the odds of asthma were 0.64 (OR = 0.64; 95% CI = 0.52, 0.78; *P* < 0.001) in the intermediate LE8 group and 0.42 (OR = 0.42; 95% CI = 0.29, 0.59; *P* < 0.001) in the high LE8 group. Similarly, the odds of chronic bronchitis were 0.73 (OR = 0.73; 95% CI = 0.55, 0.98; *P* = 0.036) in the intermediate LE8 group and 0.27 (OR = 0.27; 95% CI = 0.15, 0.49; *P* < 0.001) in the high LE8 group.

**TABLE 2:** Regression analysis of Life’s Essential 8 (LE8) and lung health.

	Model 1	Model 2	Model 3
	**OR/Beta (95% CI)** ***P*-value**	**OR/Beta (95% CI)** ***P*-value**	**OR/Beta (95% CI)** ***P*-value**
**Main cohort**			
**Asthma**			
Low (0–49)	Reference	Reference	Reference
Moderate (50–79)	0.63 (0.52, 0.77) < 0.001	0.62 (0.50, 0.76) < 0.001	0.64 (0.52, 0.78) < 0.001
High (80–100)	0.44 (0.32, 0.62) < 0.001	0.39 (0.28, 0.55) < 0.001	0.42 (0.29, 0.59) < 0.001
**Chronic bronchitis**			
Low (0–49)	Reference	Reference	Reference
Moderate (50–79)	0.53 (0.40, 0.70) < 0.001	0.71 (0.52, 0.95) 0.023	0.73 (0.55, 0.98) 0.036
High (80–100)	0.16 (0.09, 0.27) < 0.001	0.25 (0.14, 0.45) < 0.001	0.27 (0.15, 0.49) < 0.001
**Respiratory symptom sub-cohort**			
**Cough**			
Low (0–49)	Reference	Reference	Reference
Moderate (50–79)	0.39 (0.28, 0.54) < 0.001	0.43 (0.29, 0.64) < 0.001	0.44 (0.30, 0.63) < 0.001
High (80–100)	0.20 (0.12, 0.35) < 0.001	0.22 (0.11, 0.43) < 0.001	0.23 (0.12, 0.46) < 0.001
**Bringing up phlegm**			
Low (0–49)	Reference	Reference	Reference
Moderate (50–79)	0.42 (0.31, 0.58) < 0.001	0.49 (0.32, 0.74) 0.001	0.48 (0.31, 0.72) 0.001
High (80–100)	0.30 (0.16, 0.56) < 0.001	0.41 (0.19, 0.90) 0.028	0.42 (0.19, 0.90) 0.028
**Wheezing or whistling**			
Low (0–49)	Reference	Reference	Reference
Moderate (50–79)	0.40 (0.29, 0.54) < 0.001	0.43 (0.29, 0.63) < 0.001	0.44 (0.30, 0.65) < 0.001
High (80–100)	0.24 (0.15, 0.38) < 0.001	0.28 (0.15, 0.49) < 0.001	0.29 (0.15, 0.54) < 0.001
**Episode of hay fever**			
Low (0–49)	Reference	Reference	Reference
Moderate (50–79)	1.00 (0.74, 1.35) 0.993	0.86 (0.62, 1.18) 0.337	0.84 (0.59, 1.21) 0.348
High (80–100)	1.70 (1.05, 2.76) 0.031	1.21 (0.71, 2.05) 0.469	1.28 (0.72, 2.27) 0.381
**Spirometry sub-cohort**			
**Airway inflammation**			
Low (0–49)	Reference	Reference	Reference
Moderate (50–79)	0.84 (0.39, 1.79) 0.645	0.79 (0.34, 1.88) 0.591	0.78 (0.33, 1.81) 0.545
High (80–100)	0.94 (0.42, 2.10) 0.870	1.00 (0.40, 2.48) 0.999	0.84 (0.31, 2.29) 0.731
FVC (ml)	142 (109, 175) < 0.001	59 (39, 80) < 0.001	56 (32, 79) < 0.001
FEV1 (ml)	144 (113, 174) < 0.001	51 (35, 66) < 0.001	50 (32, 67) < 0.001
FEV1/FVC (%)	0.84 (0.52, 1.20) < 0.001	0.08 (−0.13, 0.29) 0.459	0.11 (−0.11, 0.33) 0.318

Model 1: Non-adjusted. Model 2: Adjusted for age, sex, race, marriage, education, PIR. Model 3: Adjusted for model 2 plus Family history of asthma, drinking_status, ALT, and AST.

For respiratory symptoms (cough, sputum, wheezing), weighted logistic regression revealed significant negative associations with LE8 scores. In model 3, compared to the low LE8 group, the odds of cough were 0.23 (OR = 0.23; 95% CI = 0.12, 0.46; *P* < 0.001) in the high LE8 group, sputum production 0.42 (OR = 0.42; 95% CI = 0.19, 0.90; *P* = 0.028), and wheezing 0.29 (OR = 0.29; 95% CI = 0.15, 0.54; *P* < 0.001). For hay fever, no significant association was observed in model 3 (*P* > 0.05).

As shown in Table 2, weighted linear regression revealed that LE8 was positively correlated with FVC and FEV1 (*P* < 0.05). In model 3, the LE8 score (per 10-point increase) was significantly positively correlated with FVC (ml) (Beta = 56; 95% CI = 32, 79; *P* < 0.001). Additionally, the LE8 score (per 10-point increase) was significantly positively correlated with FEV1 (ml) (Beta = 50; 95% CI = 32, 67; *P* < 0.001). In model 1, the LE8 score (per 10-point increase) was significantly positively correlated with FEV1/FVC (%) (Beta = 0.84; 95% CI = 0.52, 1.20; *P* < 0.001). However, this positive relationship disappeared in model 3 (*P* > 0.05), and the correlation between LE8 and airway inflammation was not significant (*P* > 0.05).

### 3.3 Non-linear relationship between LE8 and lung health

**FIGURE 3:**
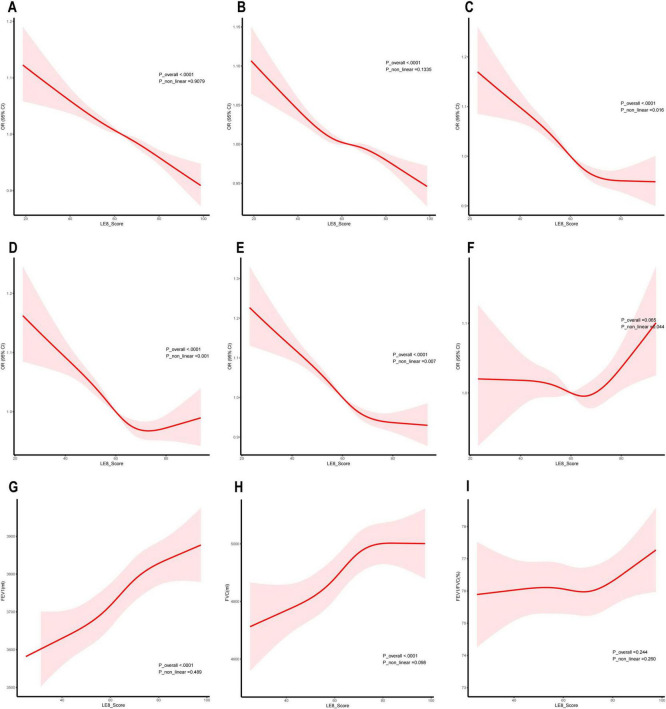
Dose-response relationship between Life’s Essential 8 (LE8) and lung health. **(A)** Asthma; **(B)** Chronic bronchitis; **(C)** Cough; **(D)** Phlegm; **(E)** Wheezing or whistling; **(F)** Hay fever; **(G)** Forced expiratory volume in one second (FEV1); **(H)** Forced vital capacity (FVC); **(I)** FEV1/FVC (%).

Figure 3 shows the restricted cubic spline (RCS) curves for lung function, lung symptoms, and lung disease, weighted by LE8. The dose-response relationship between the LE8 and various aspects of lung health is demonstrated.

For chronic lung disease, Figures 3A, B shows a linear negative correlation between the LE8 score and the prevalence of asthma and chronic bronchitis, indicating that higher LE8 scores are consistently linked with lower disease prevalence. For respiratory symptoms, Figures 3C–E shows non-linear negative correlations between LE8 scores and the prevalence of cough, sputum production, and wheezing. These curves suggest that the benefits of higher LE8 scores are most pronounced at lower score ranges, with the effects plateauing or diminishing as scores increase further. For pulmonary function, Figures 3G, H shows a linear positive link between the LE8 score and both FEV1 and FVC, indicating that better LE8 scores are consistently associated with improved lung function. Additionally, Figure 3F illustrates a non-linear relationship between LE8 scores and the prevalence of hay fever. Although the curve suggests a potential U-shaped trend, the 95% confidence intervals (CI) cross 1 across the entire range, indicating that this association is not statistically significant.

### 3.4 Subgroup analyses and sensitivity analysis

**FIGURE 4:**
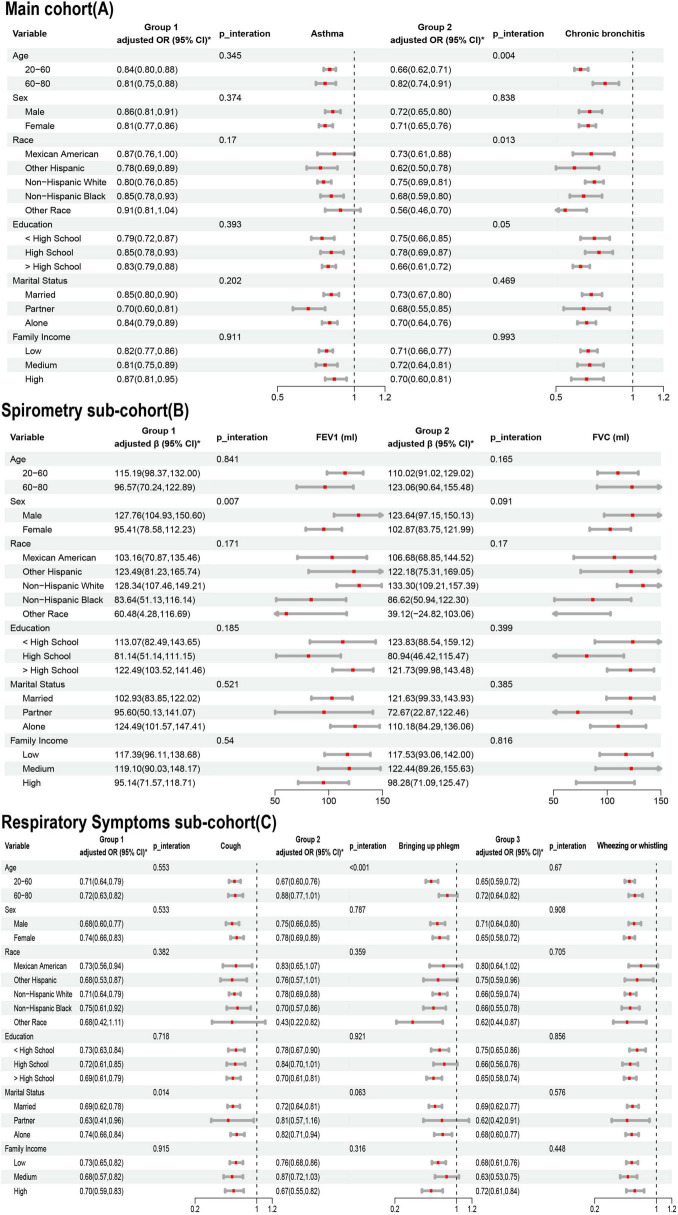
Association of Life’s Essential 8 (LE8) with lung health in age, sex, race, education, marriage, PIR subgroups. **(A)** Main cohort; **(B)** Spirometry sub-cohort; **(C)** Respiratory Symptoms sub-cohort. * Adjusted for age, sex, race, marriage, education, pir, Family history of asthma, drinking, ALT and AST.

The results of the subgroup analyses are shown in Figure 4. In the Main cohort, LE8 scores were negatively associated with asthma and chronic bronchitis. These negative correlations remained stable after stratifying by age, sex, race, education, PIR, and marital status. In the subgroup analysis for chronic bronchitis, younger individuals and participants of other races benefited more from high LE8 scores. A significant interaction was found between LE8 and both age and race in the context of chronic bronchitis (*P* < 0.05 for interaction).

In the Spirometry sub-cohort, LE8 scores were positively associated with both FEV1 and FVC, with a notable interaction between LE8 scores and FEV1. In the Respiratory Symptoms sub-cohort, LE8 scores were negatively correlated with pulmonary symptoms. For cough symptoms, there was an interaction between LE8 score and marital status. For phlegm symptoms, there was an interaction between LE8 score and age.

The results of the sensitivity analysis were robust and reliable. First, after excluding participants with missing data on key variables (LE8 and lung health) in different cohort, multiple imputation was performed for the missing covariate data. The results of the regression analysis were consistent with those of the primary analysis (Supplementary Table 5).

## 4 Discussion

To our knowledge, this is the first large-scale cross-sectional study exploring the association between LE8 and overall lung health. Initial findings indicate that higher LE8 scores are positively associated with better lung health. Subsequent models, adjusted for demographic characteristics, family history of asthma, history of alcohol consumption, and liver function, supported these findings. Specifically, the prevalence of cough, sputum production, and wheezing was lower in the high LE8 group. Additionally, lung function measures were positively correlated with LE8 scores. The prevalence of asthma and chronic bronchitis was lower in the high LE8 group compared to the low LE8 group, as confirmed by the restricted cubic spline (RCS) curves. In the subgroup analysis, these overall results remained stable.

Due to the lack of sleep assessment in Life’s Essential 7 (LE7), it is important to recognize that sleep quality is associated with many metabolic and chronic diseases. Longer sleep duration has been shown to improve daily physical activity in patients with pulmonary fibrosis (41). Conversely, shorter sleep duration predisposes individuals to asthma progression and an increased risk of emergency care, potentially mediated by low-grade inflammation and obesity (42). The inclusion of sleep quality in LE8 and its conversion to a percentile system better reflects the dose-response relationship between these factors and health outcomes. In conclusion, it is necessary to reassess the relationship LE8 and lung health to provide a more comprehensive evaluation.

The components of LE8 are well-established risk factors for lung health, so the observed correlations are not surprising. Numerous studies have supported this conclusion. For instance, Wenjun Fan, MD, and colleagues found a positive correlation between LE7 and lung function, demonstrating that adherence to ideal LE7 scores effectively reduces the prevalence of COPD in older adults (37). Another study in young adults highlighted the benefits of maintaining a healthy lifestyle for both cardiorespiratory health, emphasizing the importance of cardiopulmonary co-management (43). Disorders in lipid metabolism (44), blood glucose (13, 32), and blood pressure have all been reported to affect lung health (45), suggesting that maintaining good endocrine homeostasis is beneficial for lung health (46). Our study confirmed that the odds of asthma were 58% lower in the high LE8 group compared to the low LE8 group, and the odds of chronic bronchitis were 73% lower. Similarly, the odds of cough symptoms, sputum symptoms, and wheezing symptoms were 77, 58, and 71%, respectively, in the high LE8 group compared to the low LE8 group. Regarding lung function, every 10-point increase in LE8 was associated with a 50 ml increase in FEV1 and a 56 ml increase in FVC. These findings provide valuable insights into the association between LE8 and improved lung health.

The mechanisms underlying the link between the overall LE8 score and lung health have not yet been fully explored by scholars. However, the mechanisms involving LE8 subcomponents and lung health have been studied extensively. Nicotine exposure is known to cause the release of reactive oxygen species (ROS) with inflammatory stimuli, leading to lung tissue damage (47). In individuals with higher BMI, adipose tissue tends to be infiltrated by pro-inflammatory macrophages. These activated macrophages and hypertrophic adipocytes produce pro-inflammatory cytokines (e.g., IL-1, TNF-α, and IL-6) and adipokines, which can trigger lung discomfort (48). Additionally, obesity is often accompanied by higher insulin levels, which have been shown to induce smooth muscle hypercontractility through Phosphoinositide 3-kinase and Rho kinase-dependent pathways, as demonstrated in a bovine tracheal smooth muscle model (49). Regarding diet, the consumption of vegetables and fruits reduces airway inflammation due to their antioxidant properties (50). In contrast, excessive intake of a pro-inflammatory diet high in saturated fatty acids leads to increased ROS production and NF-κB activation (51). Dietary fiber is broken down by intestinal bacteria into metabolites that can affect airway inflammation (6, 52). It is well established that effective physical activity is key to maintaining lung health. In mouse models, physical activity has been shown to reduce airway reactivity (53).

We believe that the LE8 composite index as a valuable tool for primary prevention. As a newly developed cardiovascular health metric, LE8 is not only accessible but also provides a more holistic assessment of health compared to traditional primary care approaches that primarily focus on diet and physical activity. By integrating additional variables such as sleep, nicotine exposure, BMI, blood glucose, lipids, and blood pressure, LE8 offers a comprehensive evaluation of an individual’s overall health status. Utilizing the LE8 score in clinical practice allows for early identification of individuals at high risk for cardiorespiratory diseases and facilitates the implementation of targeted, personalized interventions. Moreover, promoting adherence to LE8-based recommendations can play a crucial role in maintaining and improving both cardiovascular and respiratory health, thereby advancing preventive healthcare at both individual and community levels. In addition, our subgroup analysis suggests that younger individuals may derive greater benefits from adherence to LE8, particularly in the context of chronic bronchitis and phlegm symptoms. Furthermore, the influence of race and sex on its effectiveness should not be overlooked. Future studies with larger populations are needed to further validate the role of LE8 across different demographic characteristics.

The present study has several strengths. To our knowledge, this is the first prospective research to investigate the connection between LE8 and lung health. We included participant data from six NHANES cycles and performed subgroup analyses based on the characteristics of the data collection. This approach allowed for a more comprehensive assessment of lung health and provided representation of a broader population. However, there were some limitations to the analysis. As a cross-sectional study, we can only draw correlations, and more cohort studies are needed to confirm causality. Another limitation of this study is the categorization of the LE8 score, a continuous variable, into predefined groups (0–49, 50–79, and 80–100). It inherently results in the loss of detailed information within the continuous range of scores and reduces the precision of association estimates by assuming homogeneity within each category. Although our study accounted for complex sampling weights to ensure representativeness, the exclusion of participants with missing data on key variables may have introduced a degree of selection bias. Additionally, lung health and lifestyle variables were self-reported, which could lead to recall bias or social desirability bias. These factors may have influenced the strength of associations observed and potentially limited the generalizability of the findings.

## 5 Conclusion

In the US population, LE8 scores were positively associated with lung health. This result suggests that promoting cardiovascular health also benefits lung health. These findings provide a valuable reference for maintaining overall cardiorespiratory health in clinical practice and primary care. Further research is needed to investigate the causal relationship and potential mechanisms between LE8 and lung health.

## Data Availability

Publicly available datasets were analyzed in this study. This data can be found here: The datasets analyzed for this study can be found in the NHANES (https://www.cdc.gov/nchs/nhanes/index.htm).
