# Supplementary material for: Association of lung health and cardiovascular health (Life’s Essential 8)
Source: Front Med (Lausanne). 2025 Feb 4;12:1481213. doi: 10.3389/fmed.2025.1481213 (PMC11844003; doi:10.3389/fmed.2025.1481213)
Supplement: Supplementary file 1 [file Supplementary_file_1.docx]

**Supplementary Table 1.** Dietary Components of Healthy Eating Index (HEI)-2015 and Scoring Standards

| **Component** | **Points Range** | **Scoring Standarda** | |
| --- | --- | --- | --- |
|  |  | **Max** | **Min** |
| HEI-2015 |  |  |  |
| Adequacy |  |  |  |
| Total Fruitsb | 0-5 | ≥0.8 cup equiv. per 1,000 kcal | 0 |
| Whole Fruitsc | 0-5 | ≥0.4 cup equiv. per 1,000 kcal | 0 |
| Total Vegetablesd | 0-5 | ≥1.1 cup equiv. per 1,000 kcal | 0 |
| Greens and Beansd | 0-5 | ≥0.2 cup equiv. per 1,000 kcal | 0 |
| Whole Grains | 0-10 | ≥1.5 oz equiv. per 1,000 kcal | 0 |
| Dairye | 0-10 | ≥1.3 cup equiv. per 1,000 kcal | 0 |
| Total Protein Foodsf | 0-5 | ≥2.5 oz equiv. per 1,000 kcal | 0 |
| Seafood and Plant Proteinse, g | 0-5 | ≥0.8 oz equiv. per 1,000 kcal | 0 |
| Fatty Acidsh HEI-2020 | 0-10 | (PUFAs + MUFAs)/SFAs ≥2.5 | (PUFAs + MUFAs)/SFAs ≤1.2 |
| Moderation |  |  |  |
| Refined Grains | 0-10 | ≤1.8 oz equiv. per 1,000 kcal | ≥4.3 oz equiv. per 1,000 kcal |
| Sodium | 0-10 | ≤1.1 grams per 1,000 kcal | ≥2.0 grams per 1,000 kcal |
| Added Sugars | 0-10 | ≤6.5% of energy | ≥26% of energy |
| Saturated Fats | 0-10 | ≤8% of energy | ≥16% of energy |

a Intakes between the minimum and maximum standards are scored proportionately. b Includes 100% fruit juice. c Includes all forms except juice. d Includes legumes (beans and peas). e Includes all milk products, such as fluid milk, yogurt, and cheese, and fortified soy beverages. F Includes legumes (beans and peas). g Includes seafood, nuts, seeds, soy products (other than beverages), and legumes (beans and peas). h Ratios of poly-and monosaturated fatty acids (PUFAs and MUFAs) to saturated fatty acids (SFAs) .

**Supplement Table 2.** Definition and scoring approach for the American Heart Association’s Life’s Essential 8 score.

| Domain | CVH Metric | Measurement | Quantification and Scoring of CVH Metric |
| --- | --- | --- | --- |
| Health Behaviors | Diet | Healthy Eating Index-2015 diet score percentile | Quantiles of Healthy Eating Index-2015 diet adherence  **Scoring (Population):**  Points Quantile  100 ≥95th percentile (top/ideal diet)  80 75th – 94th percentile  50 50th – 74th percentile  25 25th – 49th percentile  0 1st – 24th percentile (bottom/least ideal quartile) |
|  | Physical activity | Self-reported minutes of moderate or vigorous physical activity per week | Metric: Minutes of moderate (or greater) intensity activity per week  **Scoring:**  Points Minutes  100 ≥150  90 120 – 149  80 90 – 119  60 60 – 89  40 30 – 59  20 1 – 29  0 0 |
|  | Nicotine exposure | Self-reported use of cigarettes or inhaled nicotine-delivery system | Metric: Combustible tobacco use and/or inhaled NDS use; or secondhand smoke exposure  **Scoring:**  Points Status  100 Never smoker  75 Former smoker, quit ≥5 yrs  50 Former smoker, quit 1 - <5 yrs  25 Former smoker, quit <1 year, or currently using inhaled NDS  0 Current smoker  Subtract 20 points (unless score is 0) for living with active indoor smoker in home |
|  | Sleep health | Self-reported average hours of sleep per night | Metric: Average hours of sleep per night  **Scoring:**  Points Level  100 7 – <9  90 9 – <10  70 6 – <7  40 5 – <6 or ≥10  20 4 – <5  0 <4 |
| Health Factors | Body mass index | Body weight (kg) divided by height squared (m2) | Metric: Body mass index (kg/m2)  **Scoring:**  Points Level  100 <25  70 25 – 29.9  30 30 – 34.9  15 35 – 39.9  0 ≥40 |
|  | Blood lipids | Plasma total and HDL-cholesterol with calculation of non-HDL-cholesterol | Metric: Non-HDL-cholesterol (mg/dL)  **Scoring:**  Points Level  100 <130  60 130 – 159  40 160 – 189  20 190 – 219  0 ≥220  If drug-treated level, subtract 20 points |
|  | Blood glucose | Fasting blood glucose or casual hemoglobin A1c | Metric: Fasting blood glucose (mg/dL) or Hemoglobin A1c (%)  **Scoring:**  Points Level  100 No history of diabetes and FBG <100 (or HbA1c < 5.7)  60 No diabetes and FBG 100 – 125 (or HbA1c 5.7-6.4) (Pre-diabetes)  40 Diabetes with HbA1c <7.0  30 Diabetes with HbA1c 7.0 – 7.9  20 Diabetes with HbA1c 8.0 – 8.9  10 Diabetes with Hb A1c 9.0 – 9.9  0 Diabetes with HbA1c ≥10.0 |
|  | Blood pressure | Appropriately measured systolic and diastolic blood pressure | Metric: Systolic and diastolic blood pressure (mm Hg)  **Scoring:**  Points Level  100 <120/<80 (Optimal)  75 120-129/<80 (Elevated)  50 130-139 or 80-89 (Stage I HTN)  25 140-159 or 90-99  0 ≥160 or ≥100  Subtract 20 points if treated level |

**Reference:**

1. Lloyd-Jones DM, Ning H, Labarthe D, Brewer L, Sharma G, Rosamond W, Foraker RE, Black T, Grandner MA, Allen NB, et al. Status of Cardiovascular Health in US Adults and Children Using the American Heart Association’s New “Life’s Essential 8” Metrics: Prevalence Estimates From the National Health and Nutrition Examination Survey (NHANES), 2013 Through 2018. *Circulation* (2022) 146:822–835. doi: 10.1161/CIRCULATIONAHA.122.060911

2. Lloyd-Jones DM, Allen NB, Anderson CAM, Black T, Brewer LC, Foraker RE, Grandner MA, Lavretsky H, Perak AM, Sharma G, et al. Life’s Essential 8: Updating and Enhancing the American Heart Association’s Construct of Cardiovascular Health: A Presidential Advisory From the American Heart Association. *Circulation* (2022) 146:e18–e43. doi: 10.1161/CIR.0000000000001078

**Supplement Table 3:** Spirometry sub-cohort participant characterization based on LE8 scores

| Characteristic | Overall, N = 65,586,380 | Low LE8, N = 644 (13%) | Mediate LE8, N = 2717 (70%) | High LE8, N = 535 (17%) | p-value |
| --- | --- | --- | --- | --- | --- |
| Age, Years, Mean (SD) | 45.80 (15.50) | 51.48 (13.87) | 46.35 (15.48) | 39.10 (14.48) | <0.001 |
| Race(%) |  |  |  |  | <0.001 |
| Mexican American | 604 (7.4%) | 101 (8.1%) | 423 (7.4%) | 80 (6.9%) |  |
| Other Hispanic | 395 (4.5%) | 68 (5.3%) | 275 (4.5%) | 52 (4.3%) |  |
| Non-Hispanic White | 1882 (73%) | 270 (66%) | 1317 (73%) | 295 (78%) |  |
| Non-Hispanic Black | 711 (9.6%) | 177 (17%) | 493 (9.8%) | 41 (3.5%) |  |
| Other Race | 304 (5.8%) | 28 (4.1%) | 209 (5.7%) | 67 (7.4%) |  |
| Sex(%) |  |  |  |  | <0.001 |
| Male | 1971 (51%) | 327 (49%) | 1425 (54%) | 219 (39%) |  |
| Female | 1925 (49%) | 317 (51%) | 1292 (46%) | 316 (61%) |  |
| Education(%) |  |  |  |  | <0.001 |
| < High School | 821 (14%) | 192 (24%) | 566 (14%) | 63 (5.4%) |  |
| High School | 854 (20%) | 179 (29%) | 606 (21%) | 69 (11%) |  |
| > High School | 2221 (66%) | 273 (47%) | 1545 (65%) | 403 (84%) |  |
| Marital Status(%) |  |  |  |  | 0.465 |
| Married | 2099 (58%) | 314 (53%) | 1499 (58%) | 286 (58%) |  |
| Partner | 327 (8.2%) | 60 (10%) | 231 (8.2%) | 36 (6.9%) |  |
| Alone | 1470 (34%) | 270 (37%) | 987 (33%) | 213 (35%) |  |
| Family Income(%) |  |  |  |  | <0.001 |
| Low | 1594 (28%) | 342 (41%) | 1073 (28%) | 179 (20%) |  |
| Medium | 954 (25%) | 152 (27%) | 670 (25%) | 132 (25%) |  |
| High | 1348 (47%) | 150 (32%) | 974 (47%) | 224 (55%) |  |
| ALT(U/L) | 25.97 (17.20) | 28.68 (17.49) | 26.25 (17.63) | 22.69 (14.43) | <0.001 |
| AST(U/L) | 25.81 (16.53) | 26.95 (15.43) | 25.81 (17.36) | 24.91 (13.49) | 0.304 |
| Drinking(%) |  |  |  |  | <0.001 |
| Never | 1056 (22%) | 215 (30%) | 717 (22%) | 124 (18%) |  |
| Sometimes | 1783 (51%) | 232 (41%) | 1258 (51%) | 293 (62%) |  |
| Often | 1057 (26%) | 197 (29%) | 742 (28%) | 118 (20%) |  |
| FEV1 (ml) | 3,251.49 (902.61) | 2,853.71 (907.59) | 3,269.28 (900.70) | 3,484.14 (804.13) | <0.001 |
| FVC (ml) | 4,176.08 (1,104.83) | 3,733.50 (1,116.45) | 4,216.46 (1,111.82) | 4,347.97 (974.92) | <0.001 |
| FEV1/FVC (%) | 77.92 (8.05) | 76.54 (9.11) | 77.61 (7.89) | 80.29 (7.37) | <0.001 |
| LE8 | 65.96 (13.74) | 42.40 (6.08) | 65.63 (7.94) | 85.57 (4.74) | <0.001 |
| HEI‐2015 diet score |  |  |  |  | <0.001 |
| Low(0-49) | 1878 (48%) | 453 (73%) | 1321 (51%) | 104 (17%) |  |
| Moderate(50-79) | 999 (26%) | 138 (21%) | 712 (26%) | 149 (27%) |  |
| High(80-100) | 1019 (27%) | 53 (6.0%) | 684 (23%) | 282 (56%) |  |
| Physical activity score |  |  |  |  | <0.001 |
| Low(0-49) | 2284 (57%) | 542 (84%) | 1599 (58%) | 143 (27%) |  |
| Moderate(50-79) | 89 (2.3%) | 8 (0.7%) | 65 (2.3%) | 16 (3.2%) |  |
| High(80-100) | 1523 (41%) | 94 (15%) | 1053 (39%) | 376 (69%) |  |
| Sleep health score |  |  |  |  | <0.001 |
| Low(0-49) | 649 (14%) | 243 (35%) | 386 (12%) | 20 (2.3%) |  |
| Moderate(50-79) | 913 (22%) | 178 (29%) | 645 (23%) | 90 (16%) |  |
| High(80-100) | 2334 (64%) | 223 (36%) | 1686 (65%) | 425 (82%) |  |
| Nicotine exposure score |  |  |  |  | <0.001 |
| Low(0-49) | 766 (19%) | 283 (45%) | 475 (18%) | 8 (2.3%) |  |
| Moderate(50-79) | 897 (24%) | 153 (23%) | 661 (25%) | 83 (18%) |  |
| High(80-100) | 2233 (58%) | 208 (33%) | 1581 (57%) | 444 (80%) |  |
| BMI score |  |  |  |  | <0.001 |
| Low(0-49) | 1458 (35%) | 468 (74%) | 961 (34%) | 29 (4.7%) |  |
| Moderate(50-79) | 1338 (35%) | 119 (17%) | 1067 (40%) | 152 (25%) |  |
| High(80-100) | 1100 (31%) | 57 (8.8%) | 689 (26%) | 354 (70%) |  |
| Blood lipids score |  |  |  |  | <0.001 |
| Low(0-49) | 1349 (34%) | 380 (64%) | 920 (34%) | 49 (10%) |  |
| Moderate(50-79) | 963 (25%) | 124 (19%) | 732 (27%) | 107 (21%) |  |
| High(80-100) | 1584 (41%) | 140 (17%) | 1065 (38%) | 379 (69%) |  |
| Blood glucose score |  |  |  |  | <0.001 |
| Low(0-49) | 550 (9.9%) | 251 (34%) | 296 (7.6%) | 3 (0.3%) |  |
| Moderate(50-79) | 894 (19%) | 216 (35%) | 645 (20%) | 33 (4.7%) |  |
| High(80-100) | 2452 (71%) | 177 (31%) | 1776 (72%) | 499 (95%) |  |
| Blood pressure score |  |  |  |  | <0.001 |
| Low(0-49) | 840 (17%) | 329 (45%) | 497 (15%) | 14 (2.6%) |  |
| Moderate(50-79) | 1175 (30%) | 185 (31%) | 897 (34%) | 93 (16%) |  |
| High(80-100) | 1881 (52%) | 130 (24%) | 1323 (51%) | 428 (82%) |  |

**Supplement Table 4:** Respiratory Symptoms sub-cohort participant characterization based on LE8 scores

| Characteristic | Overall, N = 51,982,672 | Low LE8, N = 757 (18%) | Mediate LE8, N = 2433 (72%) | High LE8, N = 259 (10%) | p-value |
| --- | --- | --- | --- | --- | --- |
| Age, Years, Mean (SD) | 57.67 (11.61) | 58.38 (11.17) | 58.02 (11.75) | 54.01 (10.76) | <0.001 |
| Race(%) |  |  |  |  | <0.001 |
| Mexican American | 454 (5.0%) | 105 (6.0%) | 320 (4.9%) | 29 (3.5%) |  |
| Other Hispanic | 333 (3.6%) | 77 (4.7%) | 241 (3.6%) | 15 (1.7%) |  |
| Non-Hispanic White | 1847 (78%) | 347 (70%) | 1332 (78%) | 168 (87%) |  |
| Non-Hispanic Black | 632 (9.3%) | 203 (16%) | 402 (8.4%) | 27 (4.4%) |  |
| Other Race | 183 (4.3%) | 25 (3.2%) | 138 (4.6%) | 20 (3.8%) |  |
| Sex(%) |  |  |  |  | 0.056 |
| Male | 1671 (47%) | 357 (45%) | 1198 (49%) | 116 (39%) |  |
| Female | 1778 (53%) | 400 (55%) | 1235 (51%) | 143 (61%) |  |
| Education(%) |  |  |  |  | <0.001 |
| < High School | 917 (17%) | 290 (30%) | 598 (16%) | 29 (5.1%) |  |
| High School | 823 (24%) | 187 (28%) | 601 (24%) | 35 (10%) |  |
| > High School | 1709 (59%) | 280 (42%) | 1234 (60%) | 195 (85%) |  |
| Marital Status(%) |  |  |  |  | <0.001 |
| Married | 2047 (65%) | 377 (54%) | 1490 (66%) | 180 (77%) |  |
| Partner | 124 (3.7%) | 42 (6.3%) | 75 (3.0%) | 7 (3.8%) |  |
| Alone | 1278 (31%) | 338 (39%) | 868 (31%) | 72 (19%) |  |
| Family Income(%) |  |  |  |  | <0.001 |
| Low | 1421 (27%) | 426 (44%) | 931 (25%) | 64 (14%) |  |
| Medium | 858 (25%) | 177 (27%) | 623 (26%) | 58 (20%) |  |
| High | 1170 (47%) | 154 (29%) | 879 (49%) | 137 (66%) |  |
| ALT(U/L) | 25.51 (17.11) | 27.00 (22.27) | 25.20 (15.63) | 25.05 (16.60) | 0.575 |
| AST(U/L) | 26.45 (15.62) | 26.86 (18.94) | 26.16 (14.36) | 27.77 (17.61) | 0.006 |
| Drinking(%) |  |  |  |  | <0.001 |
| Never | 1272 (30%) | 318 (38%) | 887 (30%) | 67 (16%) |  |
| Sometimes | 1552 (52%) | 266 (40%) | 1126 (52%) | 160 (71%) |  |
| Often | 625 (18%) | 173 (22%) | 420 (18%) | 32 (13%) |  |
| Cough |  |  |  |  | <0.001 |
| No | 3054 (89%) | 616 (78%) | 2191 (91%) | 247 (96%) |  |
| Yes | 395 (11%) | 141 (22%) | 242 (9.5%) | 12 (4.3%) |  |
| Bringing up phlegm |  |  |  |  | <0.001 |
| No | 3098 (91%) | 641 (83%) | 2214 (92%) | 243 (95%) |  |
| Yes | 351 (9.2%) | 116 (17%) | 219 (7.8%) | 16 (5.5%) |  |
| Wheezing or whistling |  |  |  |  | <0.001 |
| No | 2959 (86%) | 570 (75%) | 2148 (88%) | 241 (93%) |  |
| Yes | 490 (14%) | 187 (25%) | 285 (12%) | 18 (7.1%) |  |
| Episode of hay fever |  |  |  |  | 0.013 |
| No | 2843 (78%) | 617 (79%) | 2033 (80%) | 193 (69%) |  |
| Yes | 606 (22%) | 140 (21%) | 400 (20%) | 66 (31%) |  |
| LE8 | 62.32 (13.62) | 41.99 (6.13) | 64.12 (8.02) | 85.29 (4.78) | <0.001 |
| HEI‐2015 diet score |  |  |  |  | <0.001 |
| Low(0-49) | 1484 (43%) | 517 (72%) | 944 (41%) | 23 (5.6%) |  |
| Moderate(50-79) | 931 (27%) | 170 (21%) | 702 (29%) | 59 (21%) |  |
| High(80-100) | 1034 (30%) | 70 (6.6%) | 787 (30%) | 177 (74%) |  |
| Physical activity score |  |  |  |  | <0.001 |
| Low(0-49) | 2203 (61%) | 648 (86%) | 1497 (60%) | 58 (26%) |  |
| Moderate(50-79) | 70 (1.8%) | 10 (0.8%) | 55 (2.0%) | 5 (2.6%) |  |
| High(80-100) | 1176 (37%) | 99 (13%) | 881 (38%) | 196 (72%) |  |
| Sleep health score |  |  |  |  | <0.001 |
| Low(0-49) | 653 (15%) | 296 (36%) | 349 (12%) | 8 (2.2%) |  |
| Moderate(50-79) | 774 (22%) | 193 (27%) | 544 (22%) | 37 (13%) |  |
| High(80-100) | 2022 (63%) | 268 (37%) | 1540 (66%) | 214 (85%) |  |
| Nicotine exposure score |  |  |  |  | <0.001 |
| Low(0-49) | 638 (18%) | 310 (44%) | 324 (14%) | 4 (0.8%) |  |
| Moderate(50-79) | 1045 (30%) | 216 (27%) | 769 (32%) | 60 (23%) |  |
| High(80-100) | 1766 (52%) | 231 (29%) | 1340 (54%) | 195 (76%) |  |
| BMI score |  |  |  |  | <0.001 |
| Low(0-49) | 1367 (37%) | 510 (67%) | 838 (34%) | 19 (5.5%) |  |
| Moderate(50-79) | 1239 (36%) | 175 (23%) | 977 (40%) | 87 (30%) |  |
| High(80-100) | 843 (26%) | 72 (9.9%) | 618 (25%) | 153 (64%) |  |
| Blood lipids score |  |  |  |  | <0.001 |
| Low(0-49) | 1408 (42%) | 441 (62%) | 927 (41%) | 40 (17%) |  |
| Moderate(50-79) | 744 (22%) | 114 (14%) | 568 (24%) | 62 (26%) |  |
| High(80-100) | 1297 (36%) | 202 (23%) | 938 (36%) | 157 (57%) |  |
| Blood glucose score |  |  |  |  | <0.001 |
| Low(0-49) | 739 (16%) | 336 (39%) | 399 (12%) | 4 (0.7%) |  |
| Moderate(50-79) | 1061 (27%) | 259 (36%) | 773 (27%) | 29 (9.2%) |  |
| High(80-100) | 1649 (57%) | 162 (24%) | 1261 (61%) | 226 (90%) |  |
| Blood pressure score |  |  |  |  | <0.001 |
| Low(0-49) | 1146 (27%) | 436 (51%) | 695 (24%) | 15 (4.9%) |  |
| Moderate(50-79) | 1063 (33%) | 181 (26%) | 813 (35%) | 69 (24%) |  |
| High(80-100) | 1240 (40%) | 140 (22%) | 925 (40%) | 175 (71%) |  |

**Supplement Table 5:** Regression analysis of LE8 and lung health after multiple imputation

|  | Model 1 | Model 2 | Model 3 |
| --- | --- | --- | --- |
|  | OR/Beta(95%CI)  P value | OR/Beta(95%CI)  P value | OR/Beta(95%CI)  P value |
| **Main cohort** |  |  |  |
| Asthma |  |  |  |
| Low(0-49) | Reference | Reference | Reference |
| Moderate(50-79) | 0.66(0.54,0.80)  <0.001 | 0.65(0.53,0.79)  <0.001 | 0.68(0.56, 0.82)  <0.001 |
| High(80-100) | 0.48(0.36,0.65)  <0.001 | 0.43(0.31,0.59)  <0.001 | 0.47(0.34,0.64)  <0.001 |
| Chronic bronchitis |  |  |  |
| Low(0-49) | Reference | Reference | Reference |
| Moderate(50-79) | 0.55(0.43,0.71)  <0.001 | 0.73(0.56,0.96)  0.024 | 0.77(0.59, 1.00)  0.046 |
| High(80-100) | 0.18(0.11,0.30)  <0.001 | 0.29(0.17,0.50)  <0.001 | 0.31(0.18,0.55)  <0.001 |
| **Respiratory symtom sub-cohort** |  |  |  |
| Cough |  |  |  |
| Low(0-49) | Reference | Reference | Reference |
| Moderate(50-79) | 0.39(0.28, 0.54)  <0.001 | 0.44(0.30, 0.63)  <0.001 | 0.45(0.32, 0.62)  <0.001 |
| High(80-100) | 0.20(0.12,0.35)  <0.001 | 0.27(0.15,0.50)  <0.001 | 0.29(0.15,0.53)  <0.001 |
| Bringing up phlegm |  |  |  |
| Low(0-49) | Reference | Reference | Reference |
| Moderate(50-79) | 0.42(0.31, 0.58)  <0.001 | 0.48(0.33, 0.69)  <0.001 | 0.48(0.34, 0.69)  <0.001 |
| High(80-100) | 0.30(0.16,0.56)  <0.001 | 0.42(0.21,0.86)  0.018 | 0.43(0.21,0.87)  0.021 |
| Wheezing or whistling |  |  |  |
| Low(0-49) | Reference | Reference | Reference |
| Moderate(50-79) | 0.40(0.29, 0.54)  <0.001 | 0.43(0.30, 0.61)  <0.001 | 0.44(0.32, 0.62)  <0.001 |
| High(80-100) | 0.24(0.15,0.38)  <0.001 | 0.26(0.16,0.44)  <0.001 | 0.29(0.17,0.48)  <0.001 |
| Episode of hay fever |  |  |  |
| Low(0-49) | Reference | Reference | Reference |
| Moderate(50-79) | 1.00(0.74, 1.35)  0.993 | 0.91(0.67, 1.24)  0.543 | 0.93(0.67, 1.28)  0.645 |
| High(80-100) | 1.70(1.05,2.76)  0.031 | 1.31(0.78,2.18)  0.296 | 1.36(0.81,2.29)  0.231 |
| **Spirometry sub-cohort** |  |  |  |
| Airway inflammation |  |  |  |
| Low(0-49) | Reference | Reference | Reference |
| Moderate(50-79) | 0.84(0.39,1.79)  0.645 | 0.86(0.37,1.98)  0.713 | 0.88(0.38,2.01)  0.747 |
| High(80-100) | 0.94(0.42,2.10)  0.870 | 1.04(0.43,2.52)  0.935 | 1.07(0.43,2.64)  0.880 |
| FVC (ml) | 142(109, 175)  <0.001 | 65(42, 89)  <0.001 | 65(39, 91)  <0.001 |
| FEV1 (ml) | 144(113, 174)  <0.001 | 56(37, 76)  <0.001 | 56(35, 78)  <0.001 |
| FEV1/FVC (%) | 0.84(0.52,1.20)  <0.001 | 0.11(-0.12,0.34)  0.340 | 0.11(-0.12,0.34)  0.340 |

Model 1: non-adjusted

Model 2 : adjusted for age, sex, race, marriage, education, pir;

Model 3 : adjusted for model 2 plus Family history of asthma, drinking_status, ALT, and AST;
